# C5L2 gene polymorphisms and their functional interaction with metabolic-inflammatory networks in T2DM-associated CHD: insights from an integrative genetic and clinical analysis in a Chinese population

**DOI:** 10.3389/fcvm.2025.1629294

**Published:** 2025-10-01

**Authors:** Mengjia Liu, Fen Liu, Dilihumaer Abulaiti, Juan Yang, Yating Wang, Meng Ye, Taotao Jia, Ying Gao

**Affiliations:** ^1^ Department of Comprehensive Internal Medicine, The First Affiliated Hospital of Xinjiang Medical University, Urumqi, Xinjiang, China; ^2^ Xinjiang Key Laboratory of Cardiovascular Disease, Clinical Medical Research Institute, The First Affiliated Hospital of Xinjiang Medical University, Urumqi, Xinjiang, China

**Keywords:** coronary heart disease (CHD), type 2 diabetes mellitus (T2DM), triglyceride-glucose index (TyG), complement 5a receptor 2 (C5L2), gene-environment interaction, multifactor dimensionality reduction (MDR)

## Abstract

**Background:**

Comorbid type 2 diabetes mellitus (T2DM) and coronary heart disease (CHD) represent a major clinical burden, driven by overlapping metabolic and inflammatory mechanisms. Genetic factors are increasingly recognized as contributors to individual susceptibility, yet the specific variants influencing T2DM-associated CHD remain incompletely defined. Complement 5a receptor 2 (C5L2) serves as a receptor for acylation-stimulating protein (ASP) and C5a, regulating glucose uptake, triglyceride clearance, lipid metabolism, and immune signaling, and has been implicated in both pro- and anti-inflammatory pathways. This study investigates whether C5L2 polymorphisms are associated with T2DM-CHD and integrate with metabolic and inflammatory markers in the Han Chinese population from Xinjiang.

**Methods:**

A hospital-based case-control study was conducted involving 951 adult participants (206 with T2DM and CHD, and 745 controls), who were genotyped for two single-nucleotide polymorphisms (SNPs), C5L2 rs2972607 and rs8112962, using improved multiplex ligation detection reaction methods. Clinical, hematologic, and biochemical traits were measured. Logistic regression assessed genotype–disease and genotype–phenotype links (sex-adjusted). MDR evaluated high-order gene–environment interactions using 10-fold cross-validation and balanced accuracy.

**Results:**

rs2972607 was significantly associated with HDL-C, lymphocytes, platelet indices, AST/ALT, UCB, and 5′-NT; rs8112962 was associated with monocytes and HDL-C. Sex-stratified analyses confirmed associations between HDL-C and UCB in both sexes; platelet effects were stronger in females. In multivariable models, rs2972607 remained a modest but significant independent predictor (OR = 2.07; *P* = 0.007). MDR identified a statistical hub comprising rs2972607 + glucose + TyG + WBC + HDL-C (Training Bal.Acc.CV = 0.996; Testing Bal.Acc.CV = 0.606; CV consistency = 10/10). These patterns align with C5L2's established roles in lipid/glucose handling and complement-driven inflammation

**Conclusions:**

C5L2 polymorphisms, particularly rs2972607, are modest but consistent contributors to T2DM–associated CHD and integrate with lipid, platelet, and inflammatory markers, highlighting a potential role in immune–metabolic interplay. While our findings are observational and hypothesis-generating, they are biologically plausible and align with established C5L2 biology, suggesting that integrating C5L2 genotyping with biochemical profiling may refine individualized risk prediction and guide future mechanistic studies.

## 1 Introduction

Coronary heart disease (CHD) is an ischemic condition arising from the narrowing or occlusion of the coronary arteries and remains the leading cause of cardiovascular disease-related mortality on a global scale. The World Heart Report 2023 indicates that cardiovascular diseases claimed approximately 20.5 million lives in 2021, accounting for nearly one-third of all deaths (1). At the same time, the China Cardiovascular Health and Disease Report 2023 highlights that cardiovascular diseases continue to be the primary cause of death among both urban and rural populations in China, with an estimated 11.39 million CHD cases, thereby underscoring the profound challenges in disease prevention and control (2).

Diabetes mellitus (DM), particularly type 2 diabetes mellitus (T2DM), is closely linked to CHD through shared pathophysiological pathways. T2DM is predominantly characterized by insulin resistance, a condition that substantially elevates the risk of developing CHD (3). Chronic hyperglycemia associated with T2DM causes a cascade of deleterious effects, most notably, vascular endothelial damage, oxidative stress, inflammation, and metabolic dysregulation, which collectively accelerate atherosclerosis and thereby heighten CHD risk (4).

Recent studies have drawn attention to the role of Complement 5a receptor 2 (C5L2), also known as C5aR2, in orchestrating inflammatory and metabolic processes. C5L2 serves as a receptor for both ASP and complement C5a, thereby regulating glucose uptake, triglyceride clearance, and lipid metabolism (5). These findings provide biological plausibility that C5L2 variants may contribute to T2DM-associated CHD by influencing metabolic, inflammatory, and thrombo-inflammatory pathways (6, 7). In addition, biomarkers like the platelet-lymphocyte ratio (PLR), the atherogenic index of plasma (AIP), and the triglyceride-glucose index (TyG) have emerged as valuable indicators of metabolic and inflammatory status. These indices correlate strongly with the progression of both T2DM and CHD, thereby offering promise in improving disease prediction and evaluation (8–10).

The development of CHD and T2DM is influenced by a variety of factors, including lifestyle choices, psychological stress, and genetic predisposition. However, research exploring the role of genetic variants and their contribution to the risk of these interrelated conditions is still limited. A seminal study conducted in 2017 identified a potential association between CHD and two single-nucleotide polymorphisms (SNPs), rs2972607 and rs8112962, within the C5L2 gene in a Chinese Han population (11). Although these variants have been implicated in CHD, their precise role in the combined pathogenesis of T2DM and CHD remains underexplored. Our study advances this understanding by specifically examining their genotype–phenotype associations and interaction with metabolic-inflammatory markers in a comorbid disease setting.

## 2 Materials and methods

### 2.1 Study population

This hospital-based case–control study matched participants by age and sex in a 1:3 ratio (cases to controls). Sample size was estimated using a literature-based prevalence (P₀ = 0.046), an expected odds ratio of 2.6, *α* = 0.05, and 90% power (*β* = 0.10), yielding a minimum requirement of 156 cases and 468 controls. The target sample was increased by 30% to reduce bias, resulting in 206 cases and 745 controls. From January 2021 to June 2024, 951 unrelated Chinese adults were consecutively recruited from the First Affiliated Hospital of Xinjiang Medical University. The case group included 206 patients (124 men, 82 women; mean age 58 ± 13 years) with both coronary heart disease (CHD) and type 2 diabetes mellitus (T2DM). The control group comprised 745 participants (494 men, 251 women; mean age 56 ± 15 years) without CHD or T2DM. All participants underwent coronary angiography, independently reviewed by two cardiologists. CHD was defined as ≥50% luminal stenosis in any major coronary artery or branch. T2DM diagnosis required clinical symptoms plus one: FPG ≥7.0 mmol/L, glucose ≥11.1 mmol/L, HbA1c ≥6.5%, random glucose ≥11.1 mmol/L, or documented history. Patients with aortic coarctation, severe heart failure, cardiogenic shock, malignant arrhythmias, renal/liver disease, endocrine or metabolic disorders, malignancies, or autoimmune diseases were excluded. To assess potential sex imbalance across genotypes, we compared the male/female distribution within rs2972607 and rs8112962 strata, and no significant differences were detected. Thus, downstream genotype–phenotype associations are unlikely to be driven by unequal sex representation. All participants gave written informed consent. The study followed the Declaration of Helsinki and was approved by the Ethics Committee of the First Affiliated Hospital of Xinjiang Medical University (Approval No. 240424-14, K202309-08).

### 2.2 Blood sample collection and hematological analysis

All participants provided informed consent and completed a demographic questionnaire that captured their age, sex, and smoking history. After an overnight fast (from 8 p.m.), 2 ml of venous blood was collected the next morning into EDTA tubes, centrifuged at 3,000 rpm for 15 min, and the plasma was stored at −80°C for further analysis. All assays were performed using standardized protocols at the Laboratory Center of Xinjiang Medical University's First Affiliated Hospital. Coagulation parameters were measured using an automated coagulation analyzer (CS-5100, Sysmex, Japan), and routine hematological indices were assessed with an automated blood cell analyzer (CAL8000, Myriad, China). The clinical panel included RBC, Hb, WBC, lymphocytes (LY), monocytes (MONO), eosinophils (EOS), basophils (BASO), LDH, PCV, MCV, MCH, RDW, PLT, MPV, PT, and APTT. Biochemical markers included glucose, triglycerides (TG), total cholesterol (TC), HDL-C, LDL-C, apolipoprotein A (apo-A), apolipoprotein B (apo-B), lipoprotein(a), and blood urea nitrogen (BUN). Several derived indices were calculated: atherogenic index of plasma [AIP = log(TG/HDL-C)], triglyceride-glucose index {TyG = ln[(TG × FPG)/2]}, systemic inflammatory response index (SIRI = NEUT × MONO/LY), systemic immune-inflammation index (SII = PLT  × NEUT/LY), neutrophil-to-lymphocyte ratio (NLR = NEUT/LY), platelet-to-lymphocyte ratio (PLR = PLT/LY), and basophil-to-albumin ratio (BAR = BUN/albumin). This approach ensured a comprehensive evaluation of hematological and biochemical status using consistent, validated methods.

### 2.3 Genomic DNA extraction and SNP genotyping

Following hospital admission, 3 ml of peripheral venous blood was aseptically collected from each participant into EDTA-containing tubes. Leukocytes were promptly isolated, and genomic DNA was extracted using the standard phenol–chloroform method. Isolated leukocytes were stored at −80°C for future analyses.

Two C5L2 SNPs (rs2972607 and rs8112962) were selected based on minor allele frequency (MAF ≥ 0.05) and linkage disequilibrium (*r*² ≥ 0.8) in the HapMap Phase II Han Chinese in Beijing (CHB) as assessed using Haploview 4.2 (11). These SNPs were prioritized because they were previously associated with coronary artery disease in a Chinese Han cohort (12), and C5L2 variants have been linked to T2DM susceptibility in independent populations (13). All participants self-reported Han Chinese ethnicity. Genotypes conformed to the Hardy-Weinberg equilibrium in cases and controls (*P* ≥ 0.05), minimizing the likelihood of population stratification. Genotyping was performed using the improved Multiplex Ligation Detection Reaction (iMLDR) technique (Genesky Biotechnologies Inc., Shanghai, China). Primer sequences are provided in Supplementary Table S1. All procedures were blinded, and 10% of samples were randomly re-genotyped for quality control. Genotypes were classified as AA, GA, GG (rs2972607) and TT, CT, CC (rs8112962).

### 2.4 Statistical analysis

All statistical analyses were performed using SPSS 26.0. Normally distributed data are presented as mean ± SD and compared using independent samples *t*-tests; non-normally distributed data are shown as median (IQR) and analyzed via rank sum and Mann–Whitney tests. Categorical variables were summarized as *n* (%) and assessed using chi-square tests, which were also applied to evaluate genotypic and allelic distributions and Hardy–Weinberg equilibrium. Associations between C5L2 polymorphisms and disease risk were examined using logistic regression, with adjusted odds ratios (OR) and 95% confidence intervals (95%CI). Given sex-specific reference ranges for HDL-C, platelet indices (PLT), and bilirubin (UCB), all regression models included sex as a covariate. In addition, we conducted prespecified sex-stratified *sensitivity checks* for HDL-C, PLT, and UCB; these supported the same association directionality observed in the primary models. Forest plots of multivariable logistic regression were generated in GraphPad Prism 9.5.0. Gene–environment interactions were analyzed using MDR (3.0.2). The dataset was partitioned via 10-fold cross-validation (10-CV), with 90% of data forming the training set and 10% serving as an independent test set. Balanced accuracy (Bal.Acc.), defined as (sensitivity + specificity)/2, was used as the performance metric, as it accounts for case–control imbalance. Training Bal.Acc.CV was the mean balanced accuracy on training folds; Testing Bal.Acc.CV reflected generalizability to independent test folds. Cross-validation consistency (CV Consistency) was the number of times a model was chosen as best across all folds. Final model selection required a high Training Bal.Acc.CV (>0.7), Testing Bal.Acc.CV ≥0.6 and close to training values, and CV Consistency >80%. All tests were two-sided, with significance set at *P* < 0.05.

### 2.5 Methodological workflow

**Figure 1:**
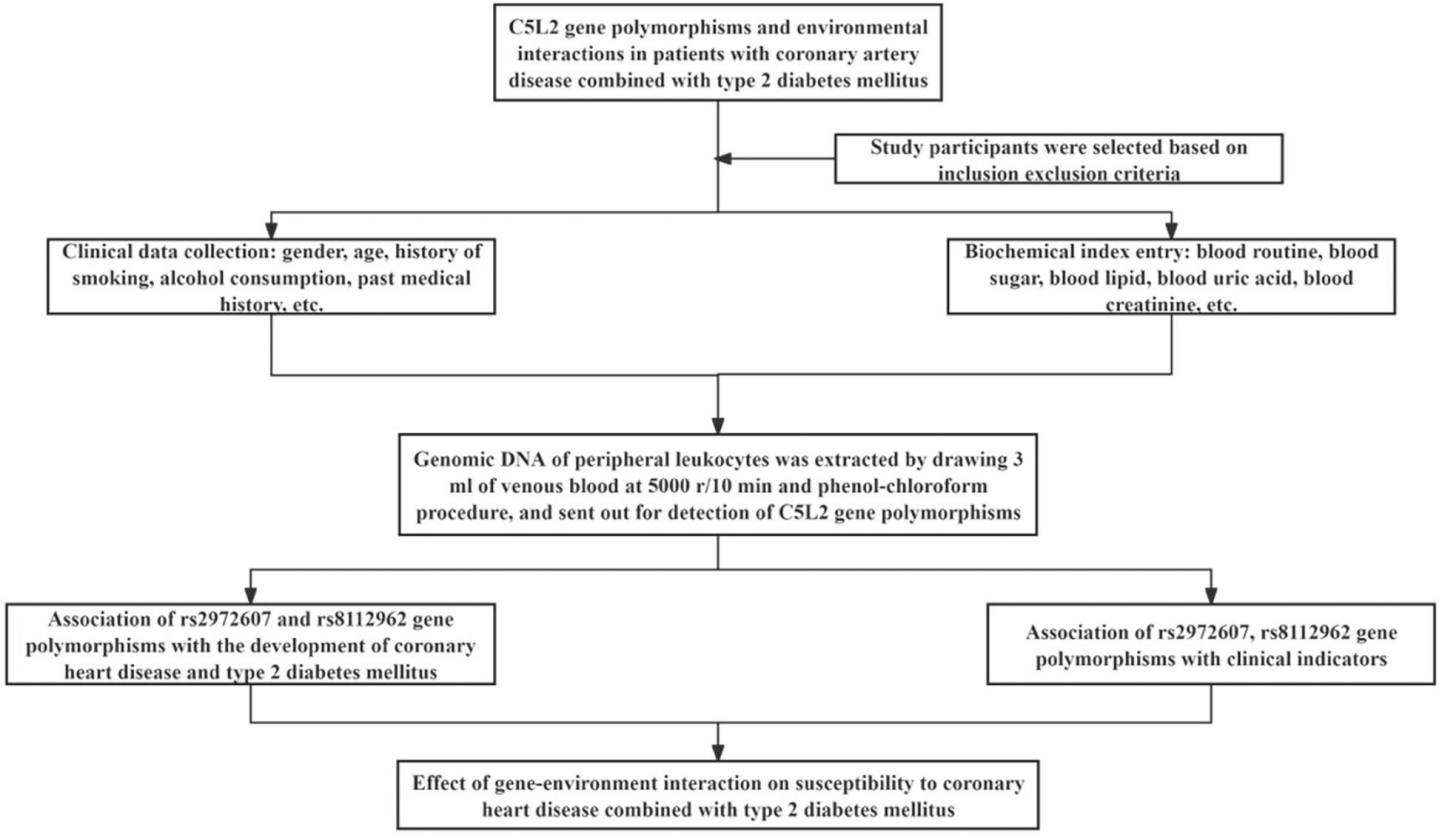
Study workflow for integrative analysis of C5L2 polymorphisms in T2DM-associated CHD. Schematic overview of the study design. Participants with comorbid T2DM and CHD, along with matched controls, underwent clinical phenotyping and genotyping for *C5L2* SNPs (rs2972607, rs8112962). Data were analyzed using multivariate logistic regression and multifactor dimensionality reduction (MDR) to evaluate independent and interactive contributions to cardiometabolic risk.

The study employed a structured workflow that integrated clinical phenotyping, biochemical profiling, and *C5L2* genotyping (rs2972607, rs8112962) to examine the interaction between *CETP* gene polymorphisms and environmental factors in patients with coronary heart disease (CHD) and type 2 diabetes mellitus (T2DM). Participants were recruited based on established inclusion and exclusion criteria. Clinical data (age, gender, smoking, alcohol consumption, and medical history) and biochemical markers (fasting blood sugar, lipid profile, and blood pressure) were systematically collected. Genomic DNA was isolated from peripheral leukocytes using a salting-out method and analyzed for *CETP* gene variants rs708272 and rs1800775 via PCR-based genotyping. The associations between genetic variants and clinical indicators were assessed, followed by evaluation of gene-environment interactions influencing CHD susceptibility in the context of T2DM. A detailed overview of the methodological workflow is presented in Figure 1.

## 3 Results

### 3.1 Baseline characteristics of study participants across clinical groups

**Figure 2:**
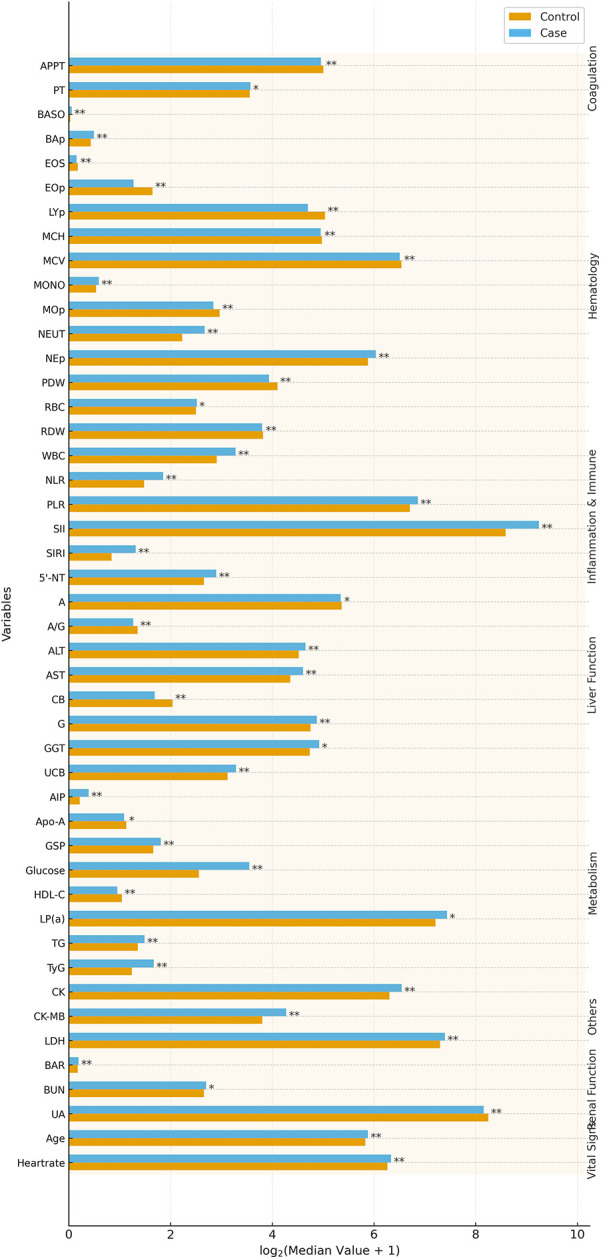
T2DM-CHD cases exhibit distinct clinical and inflammatory profiles compared to controls. Bar plots highlight significantly altered biomarkers (*P* < 0.05) in T2DM-CHD patients, reflecting systemic inflammation, metabolic dysfunction, and hepatic stress. Notable differences include elevated TyG, LDH, CK, and WBC, and reduced HDL-C and albumin. These alterations form the clinical background through which genetic associations were interpreted.

A total of 951 participants were enrolled, including 206 cases and 745 controls. The case group (124 males, 82 females; mean age 58 ± 13 years) was slightly older than the control group (494 males, 251 females; mean age 56 ± 15 years). A comparative analysis revealed significant differences (*P* < 0.05) in multiple biochemical and clinical parameters. Cases exhibited elevated levels of inflammatory and metabolic markers, including WBC, NEUT, MONO, BASO, NEp, BAp, LDH, CK, CK-MB, RBC, PT, BUN, glucose, GSP, TG, LP(a), UCB, globulin, AST, ALT, GGT, and 5′-NT. Derived indices, including AIP, SIRI, SII, TyG, NLR, BAR, and PLR, were also significantly higher, indicating heightened inflammation and metabolic dysfunction. Conversely, EOS, LYp, MOp, EOp, MCV, MCH, RDW, PDW, APTT, UA, HDL-C, Apo-A, CB, albumin, and A/G were significantly lower in the case group (*P* < 0.05), suggesting altered hematological and hepatic profiles. No significant differences were observed in sex, ethnicity, alcohol/smoking history, Hb, or PCV (*P* ≥ 0.05), indicating well-matched groups. Sex distribution did not differ across rs2972607 or rs8112962 genotype groups, supporting the validity of sex-adjusted and sex-aware inferences. A visual overview of significantly altered clinical markers is shown in Figure 2, with detailed characteristics provided in Supplementary Table S2.

### 3.2 Association of rs2972607 and rs8112962 polymorphisms with susceptibility to T2DM with coronary heart disease

**Figure 3:**
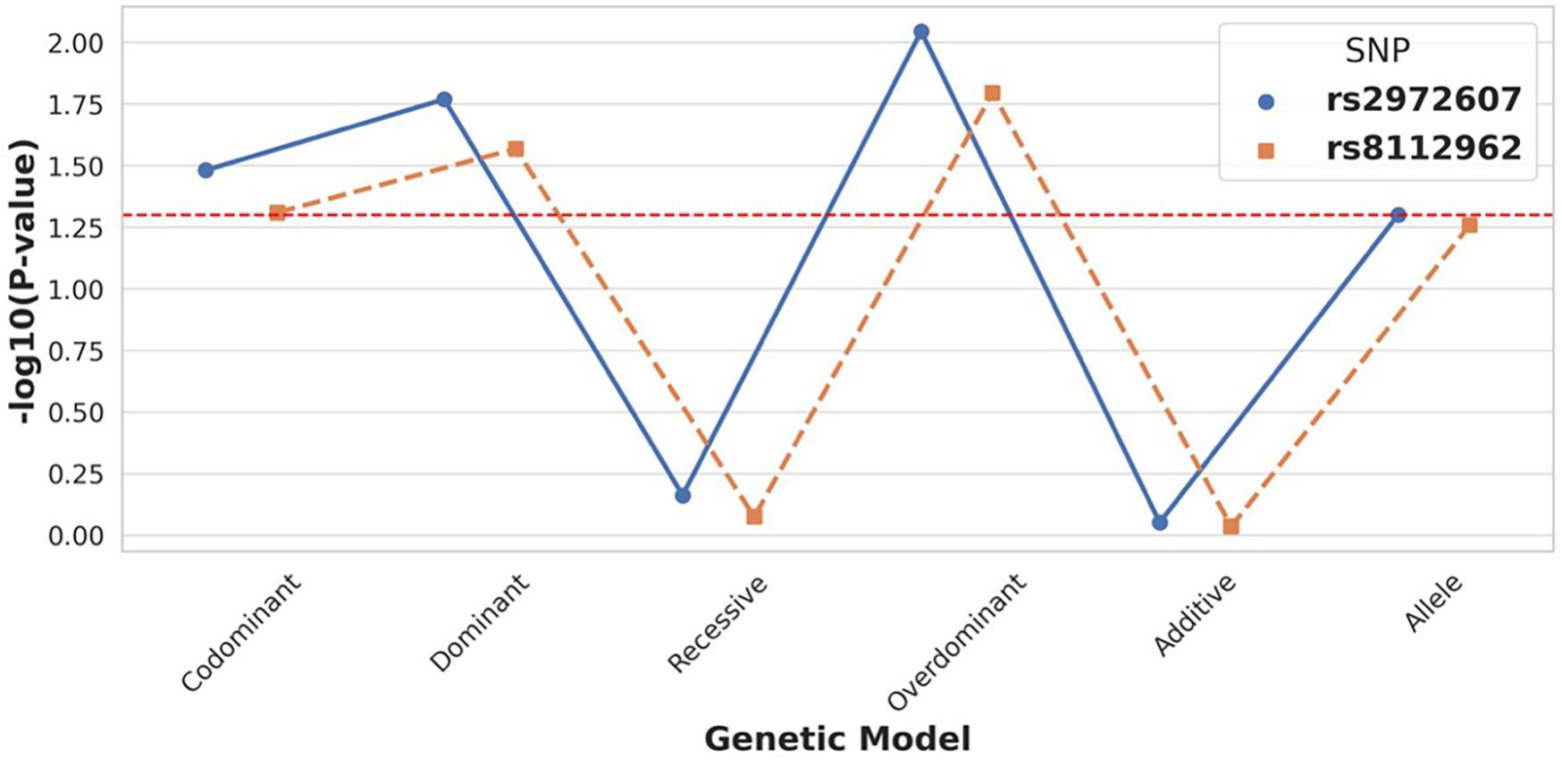
C5L2 variant rs2972607 exhibits strong association with T2DM-CHD risk under dominant and overdominant models. Comparison of genetic models for *C5L2* SNPs shows that rs2972607 is significantly associated with disease risk, particularly under dominant and overdominant models (*P* < 0.05). rs8112962 displays weaker and less consistent associations. These findings support a genotype-specific contribution of rs2972607 to CHD susceptibility in T2DM.

Genotype distributions for both rs2972607 and rs8112962 polymorphisms conformed to Hardy-Weinberg equilibrium in cases and controls (*P* ≥ 0.05), indicating genetic equilibrium and representativeness of the study population (Supplementary Figure S1, Supplementary Table S3). For rs2972607, the AA genotype was significantly underrepresented in the case group under the dominant model (AA vs. GG + GA, *P* = 0.017), and the A allele frequency was reduced compared to controls (*P* = 0.050). Conversely, the GA genotype was significantly more frequent among cases in the overdominant model (GA vs. AA + GG, *P* = 0.009), as indicated by the blue line in Figure 3. These findings suggest a protective role for the AA genotype and a potential heterozygote advantage contributing to the persistence of the G allele through balancing selection. Similarly, for rs8112962, the TT genotype was less frequent in the case group under the dominant model (TT vs. CT + CC, *P* = 0.027), indicating a protective effect. The TC genotype was significantly enriched in cases under the overdominant model (TC vs. TT + CC, *P* = 0.016), visualized by the orange line in Figure 3 (see also Supplementary Table S4). Taken together, these observations indicate that while the C allele may have deleterious effects in homozygosity. At the same time, its persistence, particularly in heterozygous form, suggests possible selective advantage, incomplete penetrance, or genetic drift. Overall, these findings support the hypothesis that genotype-specific protective or deleterious effects may vary depending on allelic combinations. Importantly, our work expands on Zheng et al. (12) by demonstrating that these polymorphisms also influence clinical traits relevant to immune function and lipid metabolism, thus providing potential biological mechanisms underlying their association with disease.

### 3.3 Association of C5L2 rs2972607 and rs8112962 genotypes with clinical and biochemical indicators

**Figure 4:**
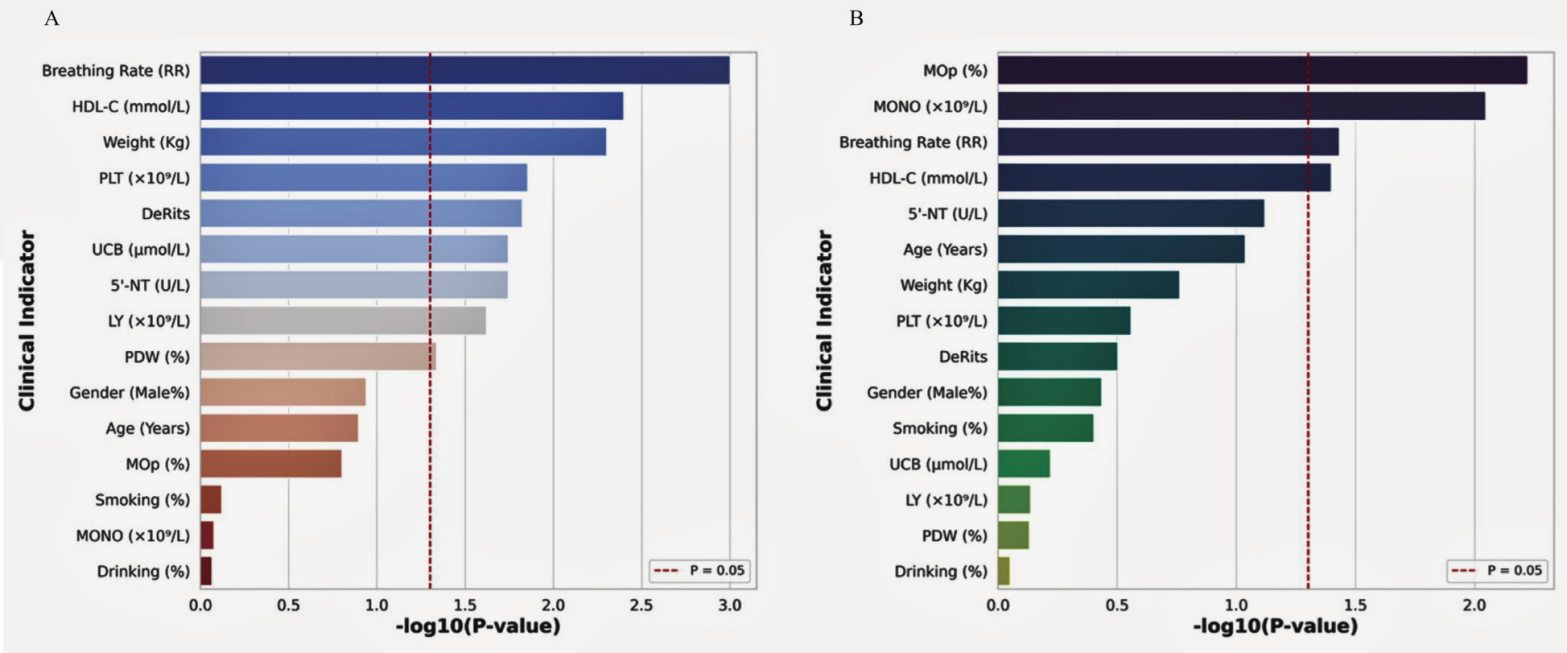
C5L2 polymorphisms show significant correlations with cardiometabolic biomarkers. Bar plots of –log₁₀ (P) values showing associations between rs2972607 **(A)** and rs8112962 **(B)** genotypes and key clinical indicators. Significant traits include HDL-C, platelet count, lymphocytes, De Ritis ratio, and 5′-nucleotidase for rs2972607; and monocytes and HDL-C for rs8112962. The dashed red line indicates a *P* < 0.05 threshold.

At the rs2972607 locus of the C5L2 gene, three genotypic variants, AA (wild-type), GA, and GG (mutant types), were identified. Similarly, at the rs8112962 locus, three genotypes were observed: TT (wild-type), CT, and CC (mutant types). Analysis of clinical parameters across subgroups of the rs2972607 genotypes revealed statistically significant differences were observed for HDL-C, PLT, AST/ALT, UCB, 5′-NT, lymphocytes, and PDW (*P* < 0.05). Because HDL-C, PLT, and UCB are sex-sensitive, we adjusted all sex models and performed prespecified sex-stratified sensitivity checks. Associations for HDL-C and UCB persisted in both sexes, while the PLT association was stronger in females. These associations are visually summarized in Figure 4A, corresponding to Supplementary Table S5. Similarly, for the rs8112962 genotypes, significant differences were observed in MOp, MONO, RR, and HDL-C across genotype subgroups (*P* < 0.05), as shown in Figure 4B and detailed in Supplementary Table S6. Despite these genotype-specific associations with biochemical and hematological markers, no statistically significant associations were found in either the rs2972607 or rs8112962 genotypes with age, gender, smoking, or alcohol consumption (Drinking) (*P* ≥ 0.05). These results underscore the functional relevance of these SNPs, suggesting that they modulate specific biochemical and inflammatory pathways rather than acting solely as passive risk markers.

### 3.4 Analysis of T2DM and CHD in the Chinese population using logistic regression

#### 3.4.1 Univariate analysis of clinical and genetic indicators

**Figure 5:**
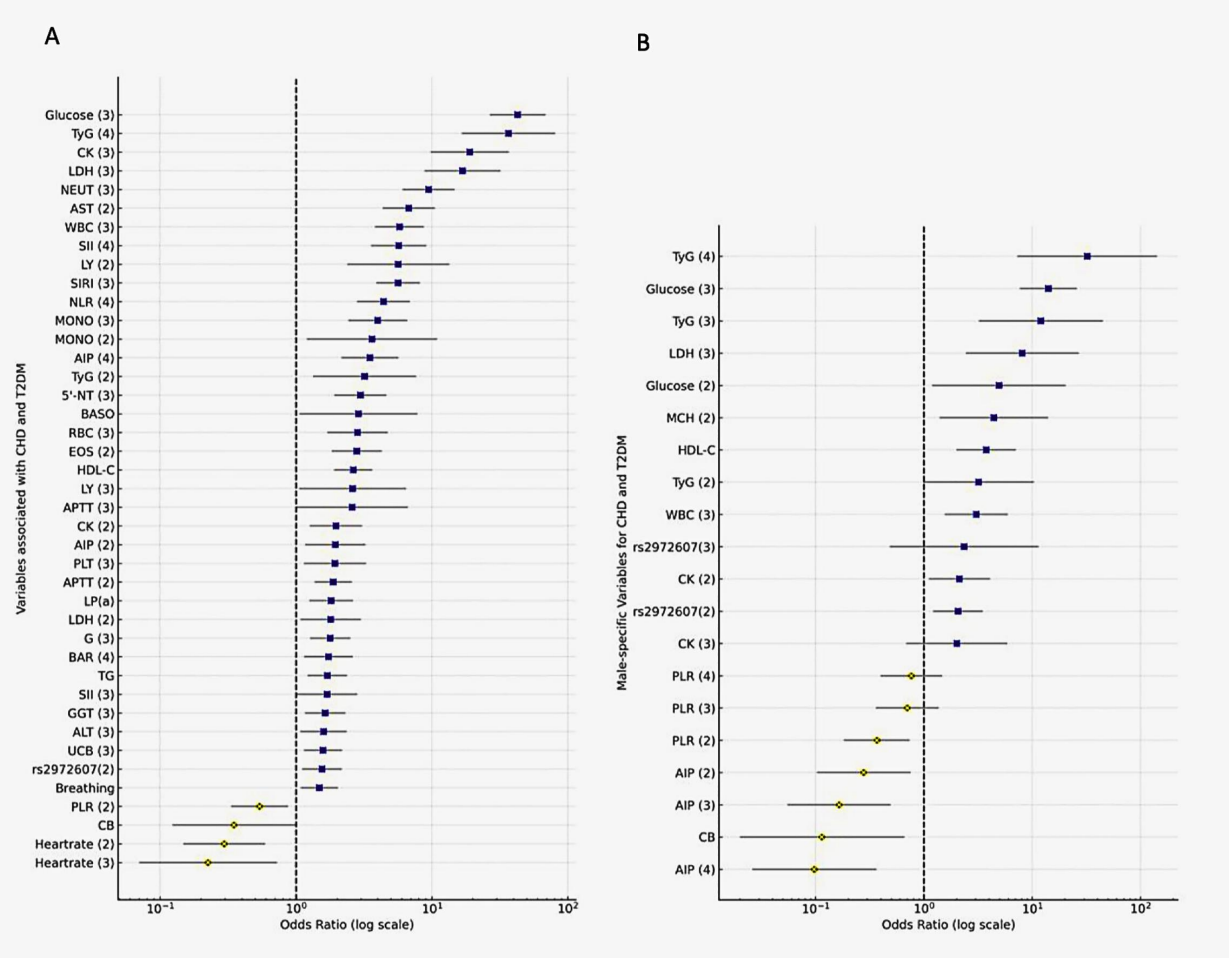
Logistic regression identifies rs2972607, glucose, and inflammatory markers as independent predictors of T2DM-CHD. Forest plots displaying odds ratios (ORs) and 95% confidence intervals from **(A)** univariate and **(B)** multivariate logistic regression analyses. Key predictors include fasting glucose, TyG index, WBC, rs2972607, and inflammatory markers. Protective indicators include PLR and HDL-C. OR >1 indicates increased risk; OR <1 indicates protective effect.

To assess the contribution of clinical and genetic factors to comorbid type 2 diabetes mellitus (T2DM) and coronary heart disease (CHD), we performed univariate logistic regression using variables categorized per the 2024 Adult Hyperuricemia and Gout Dietary Guidelines (Supplementary Table S7). As shown in Figure 5A; Supplementary Table S8, several physiological (respiratory rate, heart rate), hematological (WBC, MONO, LY, EOS, PLT), coagulation (APTT), metabolic (UA, fasting glucose, HDL-C, CB, UCB, GGT, 5'-NT), and systemic indices (AIP, SII, TyG, PLR) were significantly associated with T2DM + CHD (*P* < 0.05). Notably, the C5L2 polymorphism rs2972607 also showed a significant association (*P* = 0.034).

#### 3.4.2 Multivariate analysis identifies independent predictors

To identify independent risk factors for T2DM and CHD, variables significant in univariate analysis were included in a multivariate logistic regression model (Figure 5B; Supplementary Table S9). In multivariable models, fasting glucose was the strongest predictor (OR = 14.08; 95% CI: 7.67–25.87; *P* < 0.001), followed by WBC, MCH, HDL-C, CB, LDH, and CK. Inflammatory and metabolic markers (PLR, AIP) also remained significant, underscoring the role of systemic inflammation and metabolic dysregulation. Notably, the rs2972607 genotype remained a modest but significant independent genetic factor (OR = 2.07; *P* = 0.007). Results were robust when continuous rather than categorized predictors were used. This finding reinforces the utility of genetic screening for personalized risk stratification in patients with T2DM at risk of CHD.

### 3.5 The relationship between the C5L2 gene, environmental factors, and the onset of T2DM and CHD

**Figure 6:**
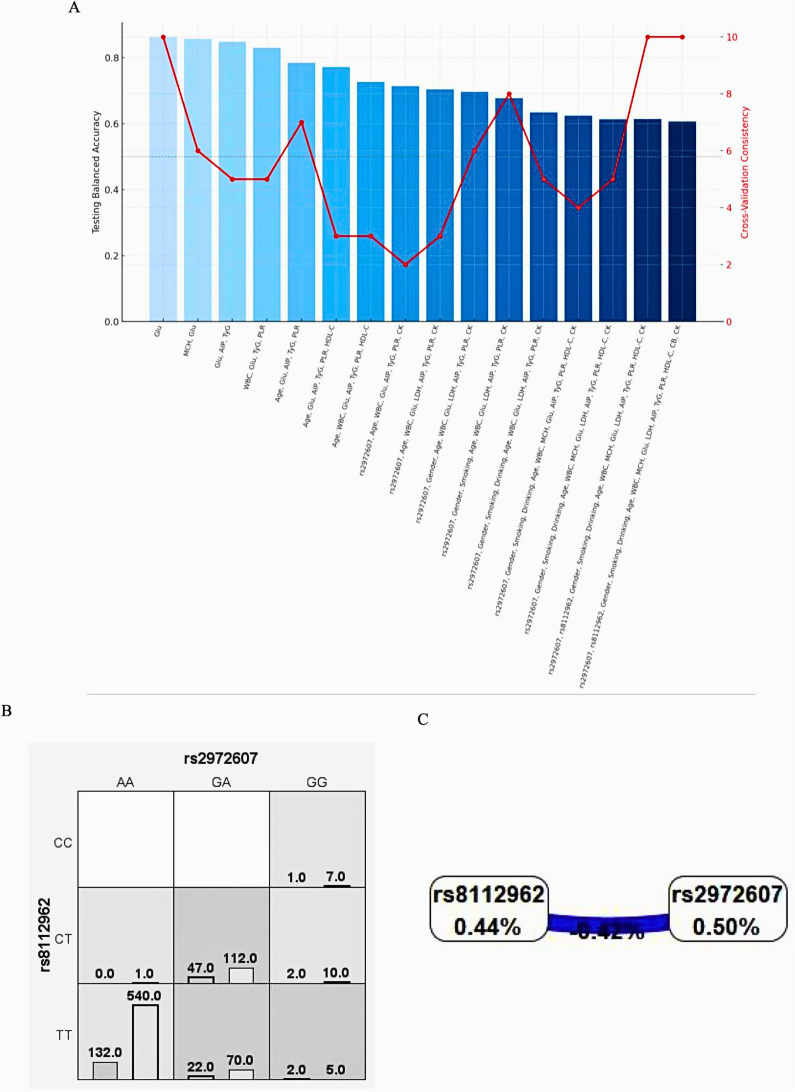
C5L2 variant rs2972607 drives high-risk genotype combinations in MDR models, while rs8112962 plays a secondary role **(A)** performance metrics of MDR models combining rs2972607 and rs8112962 with metabolic/inflammatory variables. **(B)** Case/control distribution by genotype combinations; **(C)** Dendrogram illustrating epistatic relationships and redundancy (e.g., rs2972607–rs8112962: −0.42% info gain). rs2972607 consistently emerges as the more influential variant.

To investigate the interplay between genetic and environmental factors in the pathogenesis of type 2 diabetes mellitus (T2DM) and coronary heart disease (CHD), we used multifactor dimensionality reduction (MDR) to identify high-order interactions involving C5L2 polymorphisms (rs2972607, rs8112962) and clinical/biochemical variables, as shown in Figure 6A. The best model combined these SNPs with 14 factors like gender, smoking, drinking, age, WBC, MCH, glucose, LDH, AIP, TyG, PLR, HDL-C, CB, and CK, achieving perfect cross-validation (10/10), high significance (*P* < 0.001), and strong predictive metrics (training balanced accuracy = 0.9957; testing balanced accuracy = 0.6064). Glucose consistently appeared in all top models, underscoring its central role. Notably, the addition of rs2972607 improved interaction strength, while the inclusion of rs8112962 revealed a synergistic effect, suggesting an enhanced susceptibility through combined genetic and metabolic perturbations. Although glucose alone was the best single predictor (with a balanced accuracy of 0.8633), its predictive power increased with the addition of AIP, TyG, PLR, and WBC, highlighting the multifactorial nature of the disease. The integration of these genetic variants with clinical biomarkers revealed that rs2972607 acts as a central node in metabolic-inflammatory interaction networks, particularly modulating glucose and TyG pathways, which are highly redundant yet critical predictors of cardiometabolic disease. This provides a systems-level understanding of gene–environment interplay in disease etiology. Figure 6A confirms the added value of genetic data in uncovering gene-environment interactions, while also illustrating the balance between model complexity and generalizability. This high-order model offers novel insights into T2DM with CHD and may guide future risk stratification and targeted interventions. The final model (rs2972607 + glucose + TyG + WBC + HDL-C) achieved a training balance.Acc.CV = 0.996, Testing Bal.Acc.CV = 0.606, and CV Consistency = 10/10. While this indicates strong internal stability, the modest testing accuracy reflects the complexity of polygenic disease and highlights that these results are exploratory and hypothesis-generating rather than predictive classifiers. The complete MDR model hierarchy and associated performance metrics are presented in Supplementary Table S10.

#### 3.5.1 Gene-gene interaction analyses

Multifactor Dimensionality Reduction (MDR) analysis was employed to investigate potential epistatic interactions between the C5L2 polymorphisms rs2972607 and rs8112962 and the comorbidities of type 2 diabetes mellitus (T2DM) and coronary heart disease (CHD). As shown in Figure 6B, genotype combinations were classified as high-risk (dark), low-risk (light), or neutral (white), with vertical bars representing case (left) and control (right) counts. Combinations such as TT-AA and CT-GA appeared more frequently among cases, indicating elevated risk. Figure 6C summarizes interaction effects using the information gain metric. A negative interaction information gain (–0.42%) suggests a slight redundancy, indicating that the SNPs contribute independently rather than synergistically to disease risk. Individually, rs2972607 and rs8112962 showed notable effects, with information gains of 0.50% and 0.44%, respectively, supporting their roles as independent genetic risk factors. In summary, while no strong interaction was detected, both SNPs independently contribute to CHD risk in individuals with T2DM.

#### 3.5.2 Gene-environment interaction models

**Figure 7:**
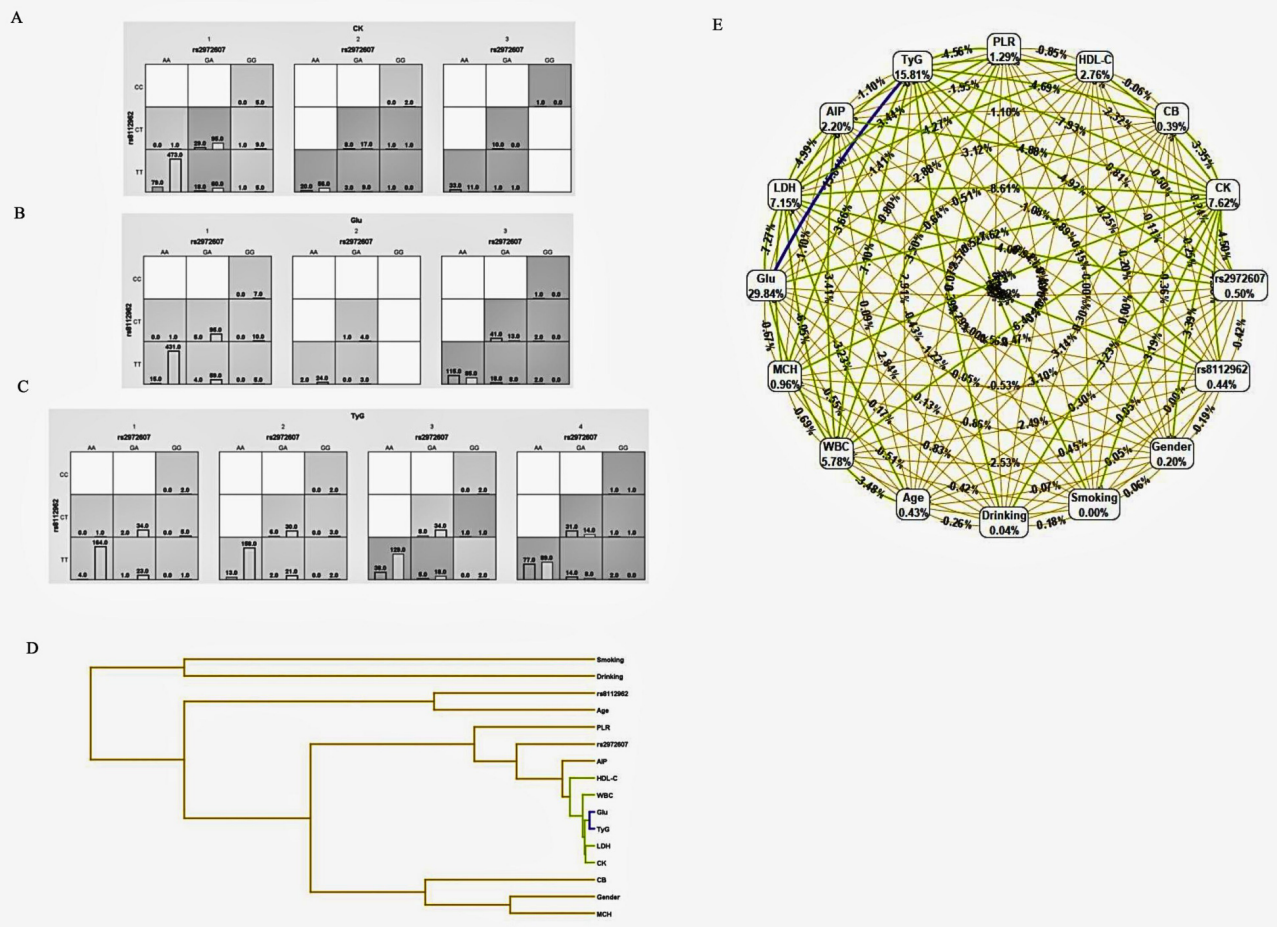
C5l2 variant rs2972607 modulates Key gene–environment interactions with glucose, TyG, and inflammatory biomarkers. MDR-based models visualizing gene–environment interactions between *C5L2* SNPs and metabolic biomarkers (e.g., CK, TyG, glucose). **(A–C)** High-risk genotype–biomarker combinations are shown via case/control bars. **(D)** Dendrogram clusters suggest synergistic and redundant interactions. **(E)** A ring network diagram quantifies main and pairwise effects, with node size and edge color denoting information gain magnitude and direction.

To explore gene-environment interactions in comorbid type 2 diabetes mellitus (T2DM) and coronary heart disease (CHD), MDR analysis was conducted. Figure 7 (Panels A–C) adopts the same color-coding as Figure 6B, classifying genotype–environment combinations into high-risk (dark grey), low-risk (light grey), and neutral (white) groups. These models assess the interactions between rs2972607 and rs8112962 in relation to clinical and biochemical markers, including creatine kinase (CK), lactate dehydrogenase (LDH), heart rate, and the triglyceride-glucose (TyG) index. In Figure 7A, rs2972607 interacts with clinical variables, e.g., the GG genotype with elevated CK or TyG is enriched in cases, indicating increased risk. Figure 7B shows similar risk associations for rs8112962. Multivariable models in Figure 7C consistently place rs2972607 in high-risk groupings, reinforcing its role in disease susceptibility. The dendrogram in Figure 7D maps variable interactions, with fasting glucose and TyG forming a strongly redundant cluster (dark blue), suggesting overlapping metabolic information. Inclusion of rs2972607 alters this redundancy, introducing weaker negative or new positive interactions (green/yellow) with WBC, LDH, CK, and HDL-C. In contrast, rs8112962 exhibits only a weak positive interaction, suggesting a more subtle effect. Overall, these results underscore the complex gene-environment interplay in cardiometabolic disease, highlighting rs2972607 as a potential mechanistic contributor through both direct and modifying effects on metabolic markers.

#### 3.5.3 Ring network diagrams of gene-environment interactions

Analysis of the gene–environment interaction network revealed that, although clinical metabolic markers dominated individual predictive power for comorbid type 2 diabetes mellitus (T2DM) and coronary heart disease (CHD), several genetic polymorphisms contributed modest but noteworthy effects, as shown in Figure 7E. In the network, each node represented a genetic or environmental factor, labeled with its information gain. At the same time, edges indicated pairwise interactions, with color and thickness reflecting the strength and direction of the interaction.

Among genetic variants analyzed, C5L2 polymorphisms rs2972607 (0.50%) and rs8112962 (0.44%) demonstrated the highest individual contributions to disease prediction within the polymorphism dataset. Their predictive value exceeded that of traditional demographic variables, such as age (0.43%) and sex (0.20%), as well as behavioral factors like alcohol consumption (0.04%). While these genetic markers contributed less than key metabolic indicators (e.g., fasting glucose: 29.84%, TyG index: 15.81%), their inclusion improved the overall model performance, particularly through interaction effects. Notably, although genetic variants showed lower individual information gain, they participated in significant interaction patterns within the network. This suggests their potential role in modulating disease susceptibility through synergistic effects with environmental or metabolic factors. For instance, some SNPs formed interaction clusters with lipid and inflammatory markers, indicating possible gene-regulatory influences on metabolic pathways relevant to cardiometabolic comorbidity. These findings underscore the importance of incorporating genetic polymorphisms into multifactorial models of disease prediction. While their standalone effects are modest, their involvement in gene–environment interactions offers valuable insight into the complex etiology of T2DM-CHD comorbidity and supports the integration of genomic data in precision medicine approaches.

## 4 Discussion

Coronary heart disease (CHD) and type 2 diabetes mellitus (T2DM) are major global health burdens with overlapping pathophysiological mechanisms. Their frequent co-occurrence heightens morbidity and mortality, yet the genetic and metabolic drivers of this comorbidity remain incompletely understood. This study investigates the role of C5L2 gene polymorphisms (rs2972607 and rs8112962) in T2DM-associated CHD, integrating clinical, inflammatory, and metabolic markers for a holistic risk model. T2DM and CHD share common mechanisms, including insulin resistance, endothelial dysfunction, oxidative stress, and chronic inflammation (7, 14, 15). Our analysis identified WBC, LDH, CK, and TyG index as strong predictors of disease risk, emphasizing their role in cardiometabolic pathology. Glucose emerged as the most informative marker, aligning with its central role in diabetic vascular complications. Previous studies also established glucose and the TyG index as predictors of CHD severity in T2DM (8, 9). Our data extend prior work by demonstrating that *C5L2* variants (particularly rs2972607) associate with lipid, platelet, and inflammatory traits and integrate into high-order interaction networks with glucose and TyG.

C5L2 encodes a G protein-coupled receptor involved in immune and metabolic regulation via ASP and C5a, exerting both pro- and anti-inflammatory effects depending on tissue context (16–24, 25, 26). While Zheng et al. (12) previously reported associations of rs2972607 and rs8112962 with CHD, our study builds upon this by establishing genotype-specific biochemical effects in the context of T2DM-CHD comorbidity and by applying advanced analytic models (MDR and logistic regression) to uncover nonlinear gene–environment interactions. Notably, rs2972607's consistent appearance in top MDR models with glucose, TyG, and HDL-C suggests it functions as a statistical hub within immune–metabolic networks, consistent with functional studies showing C5L2 regulates lipid clearance, glucose uptake, and inflammatory responses (27, 28–30). However, our study is observational and cannot establish causality, and mechanistic claims remain preliminary**.**

Our genotype–phenotype analysis revealed that rs2972607 was associated with HDL-C, platelet count, AST/ALT ratio, unconjugated bilirubin, lymphocyte count, and 5′-nucleotidase levels, suggesting its involvement in lipid transport, liver metabolism, and systemic inflammation. rs8112962 was linked with HDL-C and monocyte levels, supporting a role in immune activation (12). These functional associations provide biological plausibility for their contribution to CHD risk in the presence of T2DM. Multivariate logistic regression confirmed rs2972607 as an independent predictor, although the effect size was modest (OR ≈ 2.07), indicating that it contributes to a broader network of genetic and environmental factors. Moreover, MDR-based models demonstrated that the inclusion of rs2972607 enhanced the predictive ability of interaction networks involving glucose, TyG index, WBC, and HDL-C. These findings support a paradigm shift from isolated SNP associations toward integrative genotype–phenotype modeling, reflecting the multifactorial nature of cardiometabolic diseases. Our results also align with inflammation-based models of atherosclerosis (31–33), suggesting that C5L2 may serve as a molecular link between metabolic stress and vascular inflammation. The observed heterozygote advantage for GA (rs2972607) and CT (rs8112962) genotypes, along with the reduced frequency of AA and TT genotypes in cases, further supports the potential for balancing selection or pleiotropic effects in the evolution of complex diseases (34–37). Given that HDL-C, platelet indices, and bilirubin exhibit sex-dependent distributions, we adjusted all primary models for sex and conducted prespecified sex-stratified sensitivity checks. Associations of rs2972607 with HDL-C and UCB were consistent in both sexes, whereas the PLT signal was stronger in females. Importantly, the male/female distribution did not differ across genotype strata, minimizing the likelihood that these findings are artifacts of sex imbalance. Nonetheless, larger sex-stratified cohorts remain warranted for more precise effect estimates. Importantly, this study is the first to demonstrate the interactive impact of C5L2 SNPs on systemic indices, such as TyG and AIP, in a T2DM-CHD cohort. This systems-level approach offers new avenues for biomarker discovery and risk stratification.

At the same time, several limitations must be acknowledged. First, the retrospective, single-center design may introduce selection bias and limit generalizability to populations beyond Han Chinese. Second, we did not measure C-reactive protein (CRP), which is a widely used inflammatory biomarker. Instead, we employed systemic indices such as PLR, NLR, SII, SIRI, TyG, and AIP, which integrate hematological and metabolic markers and have been validated as robust predictors of T2DM and CHD outcomes (8, 38, 39). Third, although we minimized population stratification by restricting recruitment to Han Chinese individuals and confirming Hardy–Weinberg equilibrium, residual confounding cannot be excluded. Fourth, while MDR highlighted stable high-order interaction models, the modest testing balanced accuracy (∼0.61) suggests limited predictive power, and results should be viewed as hypothesis-generating. Finally, we did not directly measure C5L2 expression, receptor signaling, or downstream functional consequences of the SNPs. Thus, the mechanistic role of rs2972607 remains inferential, requiring future validation through CRISPR-based knockouts, complement–platelet assays, and expression profiling in cardiometabolic tissues.

In summary, our findings identify C5L2 variants, especially rs2972607, as modest but reproducible contributors to T2DM-associated CHD risk through interactions with inflammatory and metabolic pathways. By integrating genetic, biochemical, and systemic indices, this study offers a systems-level framework for hypothesis generation, while recognizing that mechanistic validation in functional models and larger, multi-ethnic cohorts will be essential to substantiate causality.

## 5 Conclusion

This study provides new insights into the genetic and metabolic mechanisms underlying the comorbidity of type 2 diabetes mellitus (T2DM) and coronary heart disease (CHD), emphasizing the clinical relevance of C5L2 gene polymorphisms. In contrast to prior studies focused solely on CHD, our work uniquely investigates the impact of rs2972607 and rs8112962 in the context of T2DM-associated CHD. We identified genotype-specific associations, particularly for rs2972607, with lipid metabolism, platelet function, and inflammatory markers. Using integrative multivariate logistic regression and multifactor dimensionality reduction (MDR) analyses, we demonstrate that rs2972607 is a modest but reproducible genetic contributor, acting as a statistical hub in immune–metabolic networks. While mechanistic claims remain preliminary, our findings highlight the value of integrating genetic screening with systemic indices for risk stratification and hypothesis generation. Our results suggest that C5L2 genotyping may enhance individualized risk stratification and inform precision prevention strategies in cardiometabolic disease.

## Data Availability

The original contributions presented in the study are included in the article/Supplementary Material, further inquiries can be directed to the corresponding author.
