# Supplementary material for: C5L2 gene polymorphisms and their functional interaction with metabolic-inflammatory networks in T2DM-associated CHD: insights from an integrative genetic and clinical analysis in a Chinese population
Source: Front Cardiovasc Med. 2025 Oct 1;12:1629294. doi: 10.3389/fcvm.2025.1629294 (PMC12521226; doi:10.3389/fcvm.2025.1629294)
Supplement: Supplementary file 1 [file Table1.docx]

**Supplementary Table 1 Primer list**

| SNP | Sequence（5’-3’） | Product（bp） |
| --- | --- | --- |
| rs2972607 | F: CAGTTGCCACTTAGGAGCATT | 210 |
|  | R: GGGAAGTTATAGCAAAACAGATGGC |  |
| rs8112962 | F: CCTCCTTAAAGCAGGTAGATTGC | 200 |
|  | R: GCAGTCACCAGCCATATGT |  |

Note: F is the forward primer, R is the reverse primer.

Notes:*,statistically significant at P＜0.05.
