# Supplementary material for: C5L2 gene polymorphisms and their functional interaction with metabolic-inflammatory networks in T2DM-associated CHD: insights from an integrative genetic and clinical analysis in a Chinese population
Source: Front Cardiovasc Med. 2025 Oct 1;12:1629294. doi: 10.3389/fcvm.2025.1629294 (PMC12521226; doi:10.3389/fcvm.2025.1629294)
Supplement: Supplementary file 2 [file Table2.docx]

**Supplementary Table S2 The baseline characteristics of controls and cases**

| Variables | Control | Case | χ²/Z | P |
| --- | --- | --- | --- | --- |
| n | 745 | 206 |  |  |
| Gender [Male%] | 494(66.3) | 124(60.2) | 2.652 | 0.103 |
| Smoking[n%] | 284(38.1) | 80(38.8) | 0.035 | 0.852 |
| Drinking[n%] | 230(30.9) | 58(28.2) | 0.564 | 0.453 |
| Age [Years (IQR)] | 56(15) | 58(13) | -3.347 | ＜0.001* |
| Heartrate [BPM, M(IQR)] | 76(13) | 80(16) | -4.264 | ＜0.001* |
| WBC [×10^9^/L, M(IQR)] | 6.51(2.11) | 8.69(3.81) | -11.141 | ＜0.001* |
| NEUT [×10^9^/L, M(IQR)] | 3.69(1.69) | 5.375(3.62) | -11.325 | ＜0.001* |
| MONO [×10^9^/L, M(IQR)] | 0.45(0.22) | 0.505(0.3) | -4.419 | ＜0.001* |
| EOS [×10^9^/L, M(IQR)] | 0.13(0.12) | 0.11(0.15) | -3.594 | ＜0.001* |
| BASO [×10^9^/L, M(IQR)] | 0.02(0.03) | 0.04(0.03) | -7.631 | ＜0.001* |
| NEp [%, M(IQR)] | 58(11.31) | 64.78(19.91) | -8.445 | ＜0.001* |
| LYp [%, M(IQR)] | 31.91(10.7) | 25.06(18.34) | -8.484 | ＜0.001* |
| MOp [%, M(IQR)] | 6.81(2.45) | 6.18(3) | -3.562 | ＜0.001* |
| EOp [%, M(IQR)] | 2.13(1.86) | 1.415(1.98) | -6.847 | ＜0.001* |
| BAp [%, M(IQR)] | 0.35(0.3) | 0.41(0.35) | -2.634 | 0.008* |
| LDH [U/L, M(IQR)] | 156.93(39) | 167.325(93.88) | -3.069 | 0.002* |
| CK [U/L, M(IQR)] | 78.2(50.3) | 92.635(183.72) | -3.625 | ＜0.001* |
| CK-MB [U/L, M(IQR)] | 13(6.48) | 18.37(24.26) | -8.604 | ＜0.001* |
| RBC [×10^12^/L, M(IQR)] | 4.66(0.7) | 4.725(0.78) | -2.035 | 0.042* |
| MCV [fl, M(IQR)] | 92.1(5.68) | 90.245(6.13) | -5.149 | ＜0.001* |
| MCH [pg, M(IQR)] | 30.5(2.2) | 30(2.1) | -4.105 | ＜0.001* |
| RDW [CV%, M(IQR)] | 13.1(0.9) | 12.95(0.8) | -3.882 | ＜0.001* |
| PDW [%, M(IQR)] | 16.2(1.72) | 14.34(4.9) | -4.925 | ＜0.001* |
| PT [s, M(IQR)] | 10.8(1.3) | 10.9(1.2) | -2.029 | 0.043* |
| APPT [s, M(IQR)] | 31.1(3.8) | 30.1(4.1) | -3.732 | ＜0.001* |
| BUN [mmol/L, M(IQR)] | 5.3(1.76) | 5.5(2.49) | -2.173 | 0.030* |
| UA [umol/L, M(IQR)] | 304(116.25) | 285.115(108.1) | -3.263 | 0.001* |
| Glucose [mmol/L, M(IQR)] | 4.89(1.02) | 10.72(7.02) | -18.906 | ＜0.001* |
| GSP [mmol/L, M(IQR)] | 2.16(0.4) | 2.5(0.79) | -8.768 | ＜0.001* |
| TG [mmol/L, M(IQR)] | 1.56(1.19) | 1.81(1.36) | -3.68 | ＜0.001 |
| HDL-C [mmol/L, M(IQR)] | 1.06(0.37) | 0.94(0.31) | -6.053 | ＜0.001* |
| Apo-A [g/L, M(IQR)] | 1.19(0.28) | 1.125(0.35) | -2.395 | 0.017* |
| LP(a) [mg/L, M(IQR)] | 147(140.86) | 172.43(210.37) | -2.146 | 0.032* |
| CB [umol/L, M(IQR)] | 3.11(2.14) | 2.225(1.85) | -7.936 | ＜0.001* |
| UCB [umol/L, M(IQR)] | 7.71(5.04) | 8.765(6.59) | -2.754 | 0.006* |
| A [g/L, M(IQR)] | 40.3(4.73) | 39.655(5.27) | -2.507 | 0.012* |
| G [g/L, M(IQR)] | 26(6) | 28.355(6.4) | -5.646 | ＜0.001* |
| A/G[M(IQR)] | 1.55(0.45) | 1.405(0.39) | -6.142 | ＜0.001* |
| AST [U/L, M(IQR)] | 19.5(8.07) | 23.4(27.42) | -5.851 | ＜0.001* |
| ALT [U/L, M(IQR)] | 22(15.61) | 24.17(20.15) | -2.693 | 0.007* |
| GGT [U/L, M(IQR)] | 25.7(21.6) | 29.39(29.9) | -2.158 | 0.031* |
| 5'-NT [U/L, M(IQR)] | 5.3(3.38) | 6.47(5.26) | -5.61 | ＜0.001* |
| AIP [M(IQR)] | 0.16(0.4) | 0.31(0.38) | -5.411 | ＜0.001* |
| SIRI [M(IQR)] | 0.789(0.665) | 1.4845(1.647) | -9.35 | ＜0.001* |
| SII [M(IQR)] | 384.24(246.83) | 606.59(756.06) | -9.089 | ＜0.001* |
| TyG [M(IQR)] | 1.363(0.819) | 2.1815(0.947) | -14.361 | ＜0.001* |
| NLR [M(IQR)] | 1.79(0.98) | 2.62(3.48) | -8.54 | ＜0.001* |
| BAR [M(IQR)] | 0.131(0.049) | 0.141(0.072) | -2.913 | 0.004* |
| PLR [M(IQR)] | 103.6(46.69) | 115.595(68.68) | -2.677 | 0.007* |

Notes：*，statistically significant at P<0.05.

Abbreviations: WBC, white blood cell count; NEUT, neutrophil count; MONO, monocyte count; EOS, eosinophil count; BASO, basophil count; NEp, neutrophil percentage; LYp, lymphocyte percentage; MOp, monocyte percentage; EOp, eosinophil percentage; BAp, basophil percentage; LDH, lactate dehydrogenase; CK, creatine kinase; CK-MB, creatine kinase isoenzyme; RBC, red blood cell count; MCV, mean corpuscular volume of red blood cells; MCH, mean corpuscular hemoglobin volume of red blood cells; RDW, red blood cell distribution width; PDW, platelet distribution width; PT, prothrombin time; APPT, activated partial thromboplastin time; BUN, blood urea nitrogen; UA, uric acid; Glucose, fasting blood glucose; GSP, glycated serum protein; TG, triglyceride; HDL-C, high-density lipoprotein cholesterol; Apo-A, apolipoprotein A; LP(a), lipoprotein (a); CB, bound bilirubin; UCB, unconjugated bilirubin; A, albumin; G, globulin; A/G, albumin/globulin ratio; AST, aspartate aminotransferase; ALT, alanine aminotransferase; GGT, gamma-glutamyl transferase; 5'-NT, 5'-nucleotidase; AIP, plasma atherosclerotic index; SIRI, systemic inflammatory response index; SII, systemic immunoinflammatory index; TyG triglyceride glucose index; NLR, neutrophil/lymphocyte ratio; BAR, basophil/albumin ratio; PLR, platelet/lymphocyte ratio.
