# Supplementary material for: C5L2 gene polymorphisms and their functional interaction with metabolic-inflammatory networks in T2DM-associated CHD: insights from an integrative genetic and clinical analysis in a Chinese population
Source: Front Cardiovasc Med. 2025 Oct 1;12:1629294. doi: 10.3389/fcvm.2025.1629294 (PMC12521226; doi:10.3389/fcvm.2025.1629294)
Supplement: Supplementary file 3 [file Table3.docx]

**Supplementary Table S3. Hardy-Weinberg balance test for two groups of patients**

| **SNP** | **Genotype** | **Control/n (%)** | | | |  | **Case/n (%)** | | | |
| --- | --- | --- | --- | --- | --- | --- | --- | --- | --- | --- |
|  |  | **Actual value** | **Theoretical value** | **χ²** | **P** |  | **Actual value** | **Theoretical value** | **χ²** | **P** |
| rs2972607 | AA | 541(72.62%) | 536(71.95%) | 0.932 | 0.628 |  | 132(64.08%) | 134(65.05%) | 0.895 | 0.639 |
|  | GA | 182(24.43%) | 192(25.77%) |  |  |  | 69(33.50%) | 64(31.07%) |  |  |
|  | GG | 22(2.95%) | 17(2.28%) |  |  |  | 5(2.42%) | 8(3.88%) |  |  |
| rs8112962 | TT | 615(82.55%) | 615(82.55%) | 0.081 | 0.960 |  | 156(75.73%) | 158(76.70%) | 1.122 | 0.595 |
|  | CT | 123(16.51%) | 124(16.64%) |  |  |  | 49(23.79%) | 45(21.84%) |  |  |
|  | CC | 7(0.94%) | 6(0.81%) |  |  |  | 1(0.48%) | 3(1.46%) |  |  |
