# Supplementary material for: C5L2 gene polymorphisms and their functional interaction with metabolic-inflammatory networks in T2DM-associated CHD: insights from an integrative genetic and clinical analysis in a Chinese population
Source: Front Cardiovasc Med. 2025 Oct 1;12:1629294. doi: 10.3389/fcvm.2025.1629294 (PMC12521226; doi:10.3389/fcvm.2025.1629294)
Supplement: Supplementary file 4 [file Table4.docx]

**Supplementary Table S4. Genotypic distribution of C5L2 polymorphisms rs2972607 and rs8112962 and their statistical comparison between case and healthy controls**

| **SNP** | | **Genotype/Allele** | **Control(*n=*745)** | **Case(*n=*206)** | ***χ*²** | ***P*** |
| --- | --- | --- | --- | --- | --- | --- |
| rs2972607 | Codominant model | AA | 541(72.6%) | 132(64.1%) | 6.854 | 0.033^*^ |
|  |  | GA | 182(24.4%) | 69(33.5%) |  |  |
|  |  | GG | 22(3.0%) | 5(2.4%) |  |  |
|  | Allele | A | 1264(84.8%) | 333(80.8%) | 3.849 | 0.050 |
|  |  | G | 226(15.2%) | 79(19.2%) |  |  |
|  | Dominant model | GG+GA | 204(27.4%) | 74(35.9%) | 5.689 | 0.017^*^ |
|  |  | AA | 541(72.6%) | 132(64.1%) |  |  |
|  | Recessive model | GG | 22(3.0%) | 5(2.4%) | 0.162 | 0.688 |
|  |  | GA+AA | 723(97.0%) | 201(97.6%) |  |  |
|  | Over dominant model | AA+GG | 563(75.6%) | 137(66.5%) | 6.827 | 0.009^*^ |
|  |  | GA | 182(24.4%) | 69(33.5%) |  |  |
|  | Additive model | AA | 541(96.1%) | 132(96.4%) | 0.020 | 0.888 |
|  |  | GG | 22(3.9%) | 5(3.6%) |  |  |
| rs8112962 | Codominant model | TT | 615(82.6%) | 156(75.7%) | 6.046 | 0.049^*^ |
|  |  | CT | 123(16.5%) | 49(23.8%) |  |  |
|  |  | CC | 7(0.9%) | 1(0.5%) |  |  |
|  | Allele | T | 1353(90.8%) | 361(87.6%) | 3.673 | 0.055 |
|  |  | C | 137(9.2%) | 51(12.4%) |  |  |
|  | Dominant model | CT+CC | 130(17.4%) | 50(24.3%) | 4.895 | 0.027^*^ |
|  |  | TT | 615(82.6%) | 156(75.7%) |  |  |
|  | Recessive model | CC | 7(0.9%) | 1(0.5%) | 0.040 | 0.841 |
|  |  | CT+TT | 738(99.1%) | 205(99.5%) |  |  |
|  | Over dominant model | TT+CC | 622(83.5%) | 157(76.2%) | 5.767 | 0.016^*^ |
|  |  | CT | 123(16.5%) | 49(23.8%) |  |  |
|  | Additive model | CC | 7(1.1%) | 1(0.6%) | 0.010 | 0.921 |
|  |  | TT | 615(98.9%) | 156(99.4%) |  |  |

Notes:*, statistically significant at *P*＜0.05.
