# Supplementary material for: C5L2 gene polymorphisms and their functional interaction with metabolic-inflammatory networks in T2DM-associated CHD: insights from an integrative genetic and clinical analysis in a Chinese population
Source: Front Cardiovasc Med. 2025 Oct 1;12:1629294. doi: 10.3389/fcvm.2025.1629294 (PMC12521226; doi:10.3389/fcvm.2025.1629294)
Supplement: Supplementary file 5 [file Table5.docx]

**Supplementary Table S5 Correlation of C5L2 gene rs2972607 genotypes with clinical** **indicators**

| **Variables** | **rs2972607 Genotype** | | | ***H/χ*²** | ***P*** |
| --- | --- | --- | --- | --- | --- |
|  | AA | GA | GG |  |  |
| *n* | 673 | 251 | 27 |  |  |
| Gender [Male%] | 63.20% | 68.50% | 77.80% | 4.321 | 0.115 |
| Smoking[n%] | 37.6% | 40.2% | 37.00% | 0.560 | 0.756 |
| Drinking[n%] | 30.60% | 29.90% | 25.90% | 0.296 | 0.862 |
| Age [Years, M(IQR)] | 57(15) | 55(14) | 51(16) | 4.132 | 0.127 |
| Weight [Kg, M(IQR)] | 74(16) | 77(16) | 73(20) | 10.623 | 0.005 |
| Breathing [RR, M(IQR)] | 19.0(2) | 19.2(2) | 19.3(2) | 14.266 | ＜0.001* |
| LY [×10^9^/L, M(IQR)] | 2.01(0.8) | 2.09(0.92) | 1.82(0.47) | 7.490 | 0.024* |
| MONO [×10^9^/L, M(IQR)] | 0.46(0.23) | 0.45(0.23) | 0.5(0.28) | 0.349 | 0.84 |
| MOp [%, M(IQR)] | 6.77(2.51) | 6.49(2.93) | 6.86(2.86) | 3.691 | 0.158 |
| PLT [×10^9^/L, M(IQR)] | 215(70) | 219(72) | 188(59) | 8.469 | 0.014* |
| PDW [%, M(IQR)] | 16.02(3.97) | 16.2(2.01) | 16.23(1.56) | 6.146 | 0.046* |
| HDL-C [mmol/L, M(IQR)] | 1.05(0.37) | 0.98(0.36) | 0.95(0.47) | 11.240 | 0.004* |
| UCB [umol/L, M(IQR)] | 8.2(4.98) | 7.38(6.26) | 5.8(4.4) | 8.069 | 0.018* |
| DeRits [M(IQR)] | 0.94(0.52) | 0.89(0.45) | 0.82(0.41) | 8.379 | 0.015* |
| 5'-NT [U/L, M(IQR)] | 5.3(3.59) | 6.1(3.6) | 6.06(5.9) | 8.020 | 0.018* |

Notes:*, statistically significant at P＜0.05.

RR is integer-valued with many ties; groups share similar medians/IQRs. The significant Kruskal–Wallis result reflects small shifts in the distribution, not large median differences

Abbreviations: LY (lymphocyte count), MONO (monocyte count), MOp (monocyte percentage), PLT (platelet count), PDW (platelet distribution width), HDL-C (high-density lipoprotein cholesterol), UCB (unconjugated bilirubin), DeRits (aspartate aminotransferase/alanine aminotransferase ratio), and 5'-NT (5'-nucleotidase).
