# Supplementary material for: C5L2 gene polymorphisms and their functional interaction with metabolic-inflammatory networks in T2DM-associated CHD: insights from an integrative genetic and clinical analysis in a Chinese population
Source: Front Cardiovasc Med. 2025 Oct 1;12:1629294. doi: 10.3389/fcvm.2025.1629294 (PMC12521226; doi:10.3389/fcvm.2025.1629294)
Supplement: Supplementary file 6 [file Table6.docx]

**Supplementary Table S6 Correlation of C5L2 gene rs8112962 genotypes with clinical indicators**

| **Variables** | **rs8112962 Genotype** | | | ***H/χ*²** | ***P*** |
| --- | --- | --- | --- | --- | --- |
|  | TT | CT | CC |  |  |
| *n* | 771 | 172 | 8 |  |  |
| Gender [Male%] | 64.50% | 66.30% | 87.50% | 2.002 | 0.368 |
| Smoking[n%] | 38 .30% | 37.20% | 62.50% | 2.049 | 0.397 |
| Drinking[n%] | 30.40% | 29.70% | 37.50% | 0.232 | 0.891 |
| Age [Years, M(IQR)] | 56(14) | 57(16) | 48(12) | 4.783 | 0.092 |
| Weight [Kg, M(IQR)] | 74(17) | 76(17) | 77(15) | 3.507 | 0.173 |
| Breathing [RR, M(IQR)] | 19(2) | 19(2) | 18.5(1) | 6.573 | 0.037* |
| LY [×10^9^/L, M(IQR)] | 2.05(0.79) | 2(0,94) | 2.28(1.21) | 0.629 | 0.730 |
| MONO [×10^9^/L, M(IQR)] | 0.47(0.24) | 0.43(0.26) | 0.49(0.15) | 9.353 | 0.009* |
| Mop [%, M(IQR)] | 6.8(2.53) | 6.24(2.8) | 6.71(2.29) | 10.282 | 0.006* |
| PLT [×10^9^/L, M(IQR)] | 215(70) | 212.5(73) | 176(116) | 2.568 | 0.277 |
| PDW [%, M(IQR)] | 16.1(3.3) | 16.12(2.49) | 16.12(1.9) | 0.603 | 0.740 |
| HDL-C [mmol/L, M(IQR)] | 1.04(0.37) | 0.98(0.38) | 1.14(0.66) | 6.434 | 0.04* |
| UCB [umol/L, M(IQR)] | 8.03(5.01) | 7.44(7.41) | 7.78(3.76) | 1.012 | 0.603 |
| DeRits [M(IQR)] | 0.92(0.52) | 0.9(0.46) | 0.7(0.45) | 2.311 | 0.315 |
| 5^'^-NT [U/L, M(IQR)] | 5.4(3.7) | 6.1(3.68) | 5.78(4.45) | 5.158 | 0.076 |

Notes:*,statistically significant at P＜0.05.

Abbreviations: LY (lymphocyte count), MONO (monocyte count), MOp (monocyte percentage), PLT (platelet count), PDW (platelet distribution width), HDL-C (high-density lipoprotein cholesterol), UCB (unconjugated bilirubin), DeRits (aspartate aminotransferase/alanine aminotransferase ratio), and 5'-NT (5'-nucleotidase).
