# Supplementary material for: C5L2 gene polymorphisms and their functional interaction with metabolic-inflammatory networks in T2DM-associated CHD: insights from an integrative genetic and clinical analysis in a Chinese population
Source: Front Cardiovasc Med. 2025 Oct 1;12:1629294. doi: 10.3389/fcvm.2025.1629294 (PMC12521226; doi:10.3389/fcvm.2025.1629294)
Supplement: Supplementary file 7 [file Table7.docx]

**Supplementary Table S7 Variable Classification and Coding Scheme for Logistic Regression Analysis**

|  | **Variables** | **Assignment** | |  | **Variables** | **Assignment** | |
| --- | --- | --- | --- | --- | --- | --- | --- |
|  |  | **Man** | **Woman** |  |  | **Man** | **Woman** |
| Y | T2DM+CHD | 0=not | | X26 | LP(a)(mg/L) | 0="＜300.00" | |
|  |  | 1=yes | |  |  | 1="≥300.00" | |
| XI | Gender | 1=Man | | X27 | CB (umol/L) | 0="＜6.80" | |
|  |  | 2=Woman | |  |  | 1="≥6.80" | |
| X2 | Smoking | 0=not | | X28 | UCB (umol/L) | 1="≥1.70&＜10.20" | |
|  |  | 1=yes | |  |  | 2="＜1.70" | |
| X3 | Drinking | 0=not | |  |  | 3="≥10.20" | |
|  |  | 1=yes | | X29 | A(g/L) | 1="≥40.00&＜55.00" | |
| X4 | Age(years) | 1="＜45" | |  |  | 2="＜40.00" | |
|  |  | 2="≥45&＜60" | |  |  | 3="≥55.00" | |
|  |  | 3="≥60" | | X30 | G(g/L) | 1="≥20.00&＜30.00" | |
| X5 | Breathing (RR) | 1="≥12&＜20" | |  |  | 2="＜20.00" | |
|  |  | 2="＜12" | |  |  | 3="≥30.00" | |
|  |  | 3="≥20" | | X31 | A/G | 1="≥1.50&＜2.50" | |
| X6 | Heartrate (BPM) | 1="≥60&＜100" | |  |  | 2="＜1.50" | |
|  |  | 2="＜60" | |  |  | 3="≥2.50" | |
|  |  | 3="≥100" | | X32 | AST(U/L) | 1="≥5.00&＜40.00" | |
| X7 | WBC(×109/L) | 1="≥4.00&＜10.00" | |  |  | 2="＜5.00" | |
|  |  | 2="＜4.00" | |  |  | 3="≥40.00" | |
|  |  | 3="≥10.00" | | X33 | ALT(U/L) | 1="≥8.00&＜40.00" | |
| X8 | NEUT(×109/L) | 1="≥2.00&＜7.00" | |  |  | 2="＜8.00" | |
|  |  | 2="＜2.00" | |  |  | 3="≥40.00" | |
|  |  | 3="≥7.00" | | X34 | GGT(U/L) | 1="≥11.00&＜50.00" | 1="≥7.00&＜32.00" |
| X9 | MONO(×109/L) | 1="≥0.12&＜0.80" | |  |  | 2="＜11.00" | 2="＜7.00" |
|  |  | 2="＜0.12" | |  |  | 3="≥50.00" | 3="≥32.00" |
|  |  | 3="≥0.80" | | X35 | 5'-NT(U/L) | 1="≥1.00&＜11.00" | |
| X10 | LY(×109/L) | 1="≥0.80&＜4.00" | |  |  | 2="＜1.00" | |
|  |  | 2="＜0.80" | |  |  | 3="≥11.00" | |
|  |  | 3="≥4.00" | | X36 | LDH(U/L) | 1="≥120.00&＜250.00" | |
| X11 | EOS(×109/L) | 1="≥0.05&＜0.50" | |  |  | 2="＜120.00" | |
|  |  | 2="＜0.05" | |  |  | 3="≥250.00" | |
|  |  | 3="≥0.50" | | X37 | CK(U/L) | 1="≥50.00&＜310.00" | 1="≥40.00&＜200.00" |
| X12 | BASO(×109/L) | 0="＜0.10" | |  |  | 2="＜50.00" | 2="＜40.00" |
|  |  | 1="≥0.10" | |  |  | 3="≥310.00" | 3="≥200.00" |
| X13 | RBC(×1012/L) | 1="≥4.00&＜5.50" | 1="≥3.50&＜5.00" | X38 | AIP | 1="＜-0.01" | |
|  |  | 2="＜4.00" | 2="＜3.50" |  |  | 2="≥-0.01&＜0.19" | |
|  |  | 3="≥5.50" | 3="≥5.00" |  |  | 3="≥0.19&＜0.40" | |
| X14 | MCV (fl) | 1="≥80.00&＜100.00" | |  |  | 4="≥0.40" | |
|  |  | 2="＜80.00" | | X39 | SIRI | 1="＜-0.561" | |
|  |  | 3="≥100.00" | |  |  | 2="≥-0.561&＜0.877" | |
| X15 | MCH (pg) | 1="≥27.00&＜34.00" | |  |  | 3="≥0.877&＜1.410" | |
|  |  | 2="＜27.00" | |  |  | 4="≥1.410" | |
|  |  | 3="≥34.00" | | X40 | SII | 1="＜288.64" | |
| X16 | RDW (%) | 1="≥11.5&＜14.5" | |  |  | 2="≥288.64&＜407.24" | |
|  |  | 2="＜11.5" | |  |  | 3="≥407.24&＜608.33" | |
|  |  | 3="≥14.5" | |  |  | 4="≥608.33" | |
| X17 | PLT(×109/L) | 1="≥100&＜300" | | X41 | TyG | 1="＜1.061" | |
|  |  | 2="＜100" | |  |  | 2="≥1.061&＜1.523" | |
|  |  | 3="≥300" | |  |  | 3="≥1.523&＜2.027" | |
| X18 | PT(s) | 1="≥11.0&＜14.0" | |  |  | 4="≥2.027" | |
|  |  | 2="＜11.0" | | X42 | NLR | 1="＜1.41" | |
|  |  | 3="≥14.0" | |  |  | 2="≥1.41&＜1.92" | |
| X19 | APTT(s) | 1="≥30.0&＜42.0" | |  |  | 3="≥1.92&＜2.66" | |
|  |  | 2="＜30.0" | |  |  | 4="≥2.66" | |
|  |  | 3="≥42.0" | | X43 | BAR | 1="＜0.109" | |
| X20 | BUN (mmol/L) | 1="≥3.20&＜7.10" | |  |  | 2="≥0.109&＜0.134" | |
|  |  | 2="＜3.20" | |  |  | 3="≥0.134&＜0.161" | |
|  |  | 3="≥7.10" | |  |  | 4="≥0.161" | |
| X21 | UA (umol/L) | 0="＜420.00" | | X44 | PLR | 1="＜84.79" | |
|  |  | 1="≥420.00" | |  |  | 2="≥84.79&＜106.37" | |
| X22 | Glucose(mmol/L) | 1="≥3.90&＜6.10" | |  |  | 3="≥106.37&＜134.09" | |
|  |  | 2="＜3.90" | |  |  | 4="≥134.09" | |
|  |  | 3="≥6.10" | | X45 | rs2972607 | 1=AA | |
| X23 | TG (mmol/L) | 0="＜2.30" | |  |  | 2=GA | |
|  |  | 1="≥2.30" | |  |  | 3=GG | |
| X24 | HDL-C(mmol/L) | 0="＞1.00" | | X46 | rs8112962 | 1=TT | |
|  |  | 1="≤1.00" | |  |  | 2=CT | |
| X25 | Apo-A(g/L) | 1="≥1.25&＜1.59" | 1="≥1.31&＜1.59" |  |  | 3=CC | |
|  |  | 2="＜1.25" | 2="＜1.31" |  |  |  | |
|  |  | 3="≥1.59" | 3="≥1.59" |  |  |  | |

Note: This table presents the categorical assignment of clinical, biochemical, and genetic variables used in logistic regression models to evaluate risk factors associated with comorbid type 2 diabetes mellitus (T2DM) and coronary heart disease (CHD). Each variable was stratified into clinically meaningful categories, with corresponding numeric codes applied for regression analysis. Genetic variants rs2972607 and rs8112962 of the **C5L2** gene were coded by genotype. Y denotes the outcome variable indicating T2DM+CHD status (0 = no, 1 = yes). **Abbreviations**: WBC (white blood cell count), NEUT (neutrophil count), MONO (monocyte count), LY (lymphocyte count), EOS (eosinophil count), and BASO (basophil count) refer to key leukocyte subsets. RBC (red blood cell count), MCV (mean corpuscular volume of red blood cells), MCH (mean corpuscular hemoglobin content of red blood cells), and RDW (red blood cell distribution width) are hematological parameters related to erythrocyte morphology and function. PLT (platelet count), PT (prothrombin time), and APTT (activated partial thromboplastin time) are indicators of coagulation. BUN (blood urea nitrogen), UA (uric acid), and Glucose (fasting glucose) are metabolic markers, while TG (triglyceride), HDL-C (high-density lipoprotein cholesterol), Apo-A (apolipoprotein A), and LP(a) [lipoprotein (a)] are lipid-related indicators. CB (bound bilirubin), UCB (unconjugated bilirubin), A (albumin), G (globulin), and A/G (albumin/globulin ratio) reflect liver and protein metabolism. Liver enzymes include AST (aspartate aminotransferase), ALT (alanine aminotransferase), GGT (gamma-glutamyl transferase), and 5'-NT (5'-nucleotidase), while LDH (lactate dehydrogenase) and CK (creatine kinase) are markers of tissue damage and cellular turnover. Composite indices include AIP (atherosclerotic index of plasma), SIRI (systemic inflammatory response index), SII (systemic immune response index), TyG (triglyceride glucose index), NLR (neutrophil/lymphocyte ratio), BAR (basophil/albumin ratio), and PLR (platelet/lymphocyte ratio), which capture systemic inflammation, immune balance, and metabolic status.
