# Supplementary material for: C5L2 gene polymorphisms and their functional interaction with metabolic-inflammatory networks in T2DM-associated CHD: insights from an integrative genetic and clinical analysis in a Chinese population
Source: Front Cardiovasc Med. 2025 Oct 1;12:1629294. doi: 10.3389/fcvm.2025.1629294 (PMC12521226; doi:10.3389/fcvm.2025.1629294)
Supplement: Supplementary file 9 [file Table9.docx]

**Supplementary Table S9 Multivariate Logistic Regression Analysis of Risk Factors for T2DM with CHD**

| **Variables** | **P value** | **OR** | **95% CI for OR** | |
| --- | --- | --- | --- | --- |
|  |  |  | **Lower** | **Upper** |
| WBC | 0.003* |  |  |  |
| WBC (2) | 0.341 | 0.317 | 0.030 | 3.375 |
| WBC (3) | 0.001* | 3.043 | 1.553 | 5.963 |
| MCH | 0.019* |  |  |  |
| MCH (2) | 0.012* | 4.425 | 1.393 | 14.057 |
| MCH (3) | 0.210 | 0.118 | 0.004 | 3.348 |
| Glucose | ＜0.001* |  |  |  |
| Glucose (2) | 0.027* | 4.935 | 1.194 | 20.394 |
| Glucose (3) | ＜0.001* | 14.082 | 7.666 | 25.870 |
| HDL-C | ＜0.001* | 3.765 | 2.002 | 7.079 |
| CB | 0.016* | 0.114 | 0.020 | 0.662 |
| LDH | 0.001* |  |  |  |
| LDH (2) | 0.256 | 0.656 | 0.317 | 1.357 |
| LDH (3) | 0.001* | 8.091 | 2.434 | 26.893 |
| CK | 0.042* |  |  |  |
| CK (2) | 0.024* | 2.127 | 1.107 | 4.088 |
| CK (3) | 0.202* | 2.012 | 0.687 | 5.895 |
| AIP | 0.006* |  |  |  |
| AIP (2) | 0.012* | 0.278 | 0.103 | 0.754 |
| AIP (3) | 0.001* | 0.165 | 0.055 | 0.494 |
| AIP (4) | 0.001* | 0.097 | 0.026 | 0.365 |
| TyG | ＜0.001* |  |  |  |
| TyG (2) | 0.053 | 3.193 | 0.984 | 10.358 |
| TyG (3) | ＜0.001* | 12.003 | 3.215 | 44.805 |
| TyG (4) | ＜0.001* | 32.178 | 7.265 | 142.527 |
| PLR | 0.041* |  |  |  |
| PLR (2) | 0.005* | 0.368 | 0.183 | 0.737 |
| PLR (3) | 0.301 | 0.702 | 0.360 | 1.371 |
| PLR (4) | 0.424 | 0.764 | 0.396 | 1.476 |
| rs2972607 | 0.021* |  |  |  |
| rs2972607(2) | 0.007* | 2.066 | 1.219 | 3.503 |
| rs2972607(3) | 0.289 | 2.352 | 0.484 | 11.438 |

Notes:*,statistically significant at P＜0.05. **Abbreviations**: WBC (white blood cell count), MCH (mean corpuscular hemoglobin content of red blood cells), Glucose (fasting glucose), HDL-C (high-density lipoprotein cholesterol), CB (bound bilirubin), LDH (lactate dehydrogenase), CK (creatine kinase), AIP (atherogenic index of plasma), TyG (triglyceride-glucose index), and PLR (platelet-lymphocyte ratio).
