# Supplementary material for: C5L2 gene polymorphisms and their functional interaction with metabolic-inflammatory networks in T2DM-associated CHD: insights from an integrative genetic and clinical analysis in a Chinese population
Source: Front Cardiovasc Med. 2025 Oct 1;12:1629294. doi: 10.3389/fcvm.2025.1629294 (PMC12521226; doi:10.3389/fcvm.2025.1629294)
Supplement: Supplementary file 10 [file Table10.docx]

**Supplementary Table S10. Multifactor Dimensionality Reduction (MDR) Models Assessing Gene–Environment Interactions Involving *C5L2* Polymorphisms in T2DM with CHD**

| **Model** | **Training Bal.Acc.CV** | **Testing Bal.Acc.CV** | **CV Consistency** | **P value** |
| --- | --- | --- | --- | --- |
| Glu | 0.8633 | 0.8633 | 10/10 | ＜0.001* |
| MCH, Glu | 0.8663 | 0.8571 | 6/10 | ＜0.001* |
| Glu, AIP, TyG | 0.8744 | 0.8491 | 5/10 | ＜0.001* |
| WBC, Glu, TyG, PLR | 0.8874 | 0.8306 | 5/10 | ＜0.001* |
| Age, Glu, AIP, TyG, PLR | 0.9077 | 0.7847 | 7/10 | ＜0.001* |
| Age, Glu, AIP, TyG, PLR, HDL-C | 0.9275 | 0.7723 | 3/10 | ＜0.001* |
| Age, WBC, Glu, AIP, TyG, PLR, HDL-C | 0.9463 | 0.7269 | 3/10 | ＜0.001* |
| rs2972607, Age, WBC, Glu, AIP, TyG, PLR, CK | 0.9627 | 0.7142 | 2/10 | ＜0.001* |
| rs2972607, Age, WBC, Glu, LDH, AIP, TyG, PLR, CK | 0.9758 | 0.7043 | 3/10 | ＜0.001* |
| rs2972607, Gender, Age, WBC, Glu, LDH, AIP, TyG, PLR, CK | 0.9839 | 0.6962 | 6/10 | ＜0.001* |
| rs2972607, Gender, Smoking, Age, WBC, Glu, LDH, AIP, TyG, PLR, CK | 0.9892 | 0.6773 | 8/10 | ＜0.001* |
| rs2972607, Gender, Smoking, Drinking, Age, WBC, Glu, LDH, AIP, TyG, PLR, CK | 0.9924 | 0.6344 | 5/10 | ＜0.001* |
| rs2972607, Gender, Smoking, Drinking, Age, WBC, MCH, Glu, AIP, TyG, PLR, HDL-C, CK | 0.9941 | 0.6243 | 4/10 | ＜0.001* |
| rs2972607, Gender, Smoking, Drinking, Age, WBC, MCH, Glu, LDH, AIP, TyG, PLR, HDL-C, CK | 0.9952 | 0.6137 | 5/10 | ＜0.001* |
| rs2972607, rs8112962, Gender, Smoking, Drinking, Age, WBC, MCH, Glu, LDH, AIP, TyG, PLR, HDL-C, CK | 0.9957 | 0.6145 | 10/10 | ＜0.001* |
| rs2972607, rs8112962, Gender, Smoking, Drinking, Age, WBC, MCH, Glu, LDH, AIP, TyG, PLR, HDL-C, CB, CK | 0.9957 | 0.6064 | 10/10 | ＜0.001* |
