# Supplementary material for: C5L2 gene polymorphisms and their functional interaction with metabolic-inflammatory networks in T2DM-associated CHD: insights from an integrative genetic and clinical analysis in a Chinese population
Source: Front Cardiovasc Med. 2025 Oct 1;12:1629294. doi: 10.3389/fcvm.2025.1629294 (PMC12521226; doi:10.3389/fcvm.2025.1629294)
Supplement: Supplementary file 11 [file Image1.pdf]

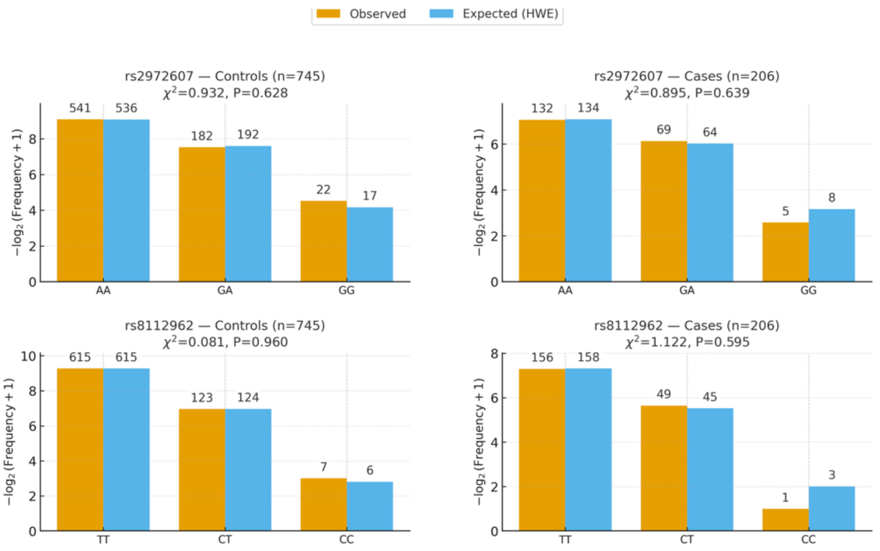

Supplementary Figure 1. Genotypic Distributions and Hardy-Weinberg Equilibrium Assessment of rs2972607 and rs8112962 in Case and Control Groups. Bar plots illustrating the actual and theoretical genotype frequencies for the single-nucleotide polymorphisms (SNPs) rs2972607 and rs8112962 in control and case groups. The y-axis represents the $\log_2$-transformed genotype. For each SNP, control groups are depicted in light blue and case groups in dark blue, with actual and Hardy-Weinberg equilibrium (HWE) theoretical values shown side-by-side for each genotype (AA, GA/CT, GG/CC). P-values from the chi-square goodness-of-fit test for HWE are displayed below each subplot's legend
